# Poland's syndrome and recurrent pneumothorax: is there a connection?

**DOI:** 10.1186/1749-8090-6-32

**Published:** 2011-03-19

**Authors:** Georgios I Tagarakis, Dimos Karangelis, Angheliki Tsantsaridou, Fani Tsolaki, Marios E Daskalopoulos, Athanassios Hevas, Katerina Kyriakaki, Konstantinos Stamoulis, Stefania Lampoura, Nikolaos B Tsilimingas

**Affiliations:** 1 Department of Cardiovascular and Thoracic Surgery, University Hospital of Thessaly, Larissa, Greece

## Abstract

Aim. To investigate the possible connection of Poland's syndrome with the presence of lung bullae and, thus, with an increased risk for recurrent pneumothorax. Patients-methods. Two male patients, aged 19 and 21 years respectively were submitted to our department after their second incident of pneumothorax. Both had Poland's syndrome (unilaterally hypoplastic chest wall with pectoralis major muscle atrophy) and both had multiple bullae to the ipsilateral lung based on CT findings. The patients were treated operatively (bullectomy, lung apicectomy, partial parietal pleurectomy and chemical pleurodesis) due to the recurrent state of their pneumothorax. Results. The patients had good results with total expansion of the affected lung. Conclusions. Poland's syndrome can be combined with ipsilateral presence of lung bullae, a common cause of pneumothorax. Whether this finding is part or a variation of the syndrome needs to be confirmed by a larger number of similar cases.

## Introduction

Poland's syndrome is a developmental deformity characterized by hypoplastic (unilaterally) chest wall with pectoralis major muscle atrophy, sometimes combined with syndactyly. It has an incidence of 1:7000 to a: 100000 live births and affects with a higher frequency (2:1) the right hemithorax [1,2]. It was first described by British Alfred Poland [3] and about 100 years later studied by the British doctor Patrick Clarkson [2] who named it after him. The syndrome is in rare cases combined with severe ocular pathologies, such as hamartoma of the retina and retinal pigment epithelium [4]. Herein, we are presenting two case reports of patients with the syndrome which was combined with bullae of the ipsilateral lung and suffered from recurrent episodes of pneumothorax.

## Cases Presentation

**Figure 1:**
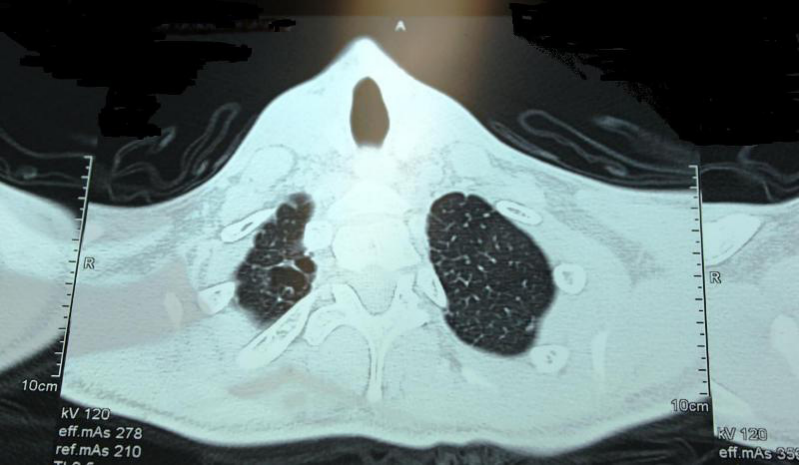
**CT Image with the bullae of the right lung apex**.

**Figure 2:**
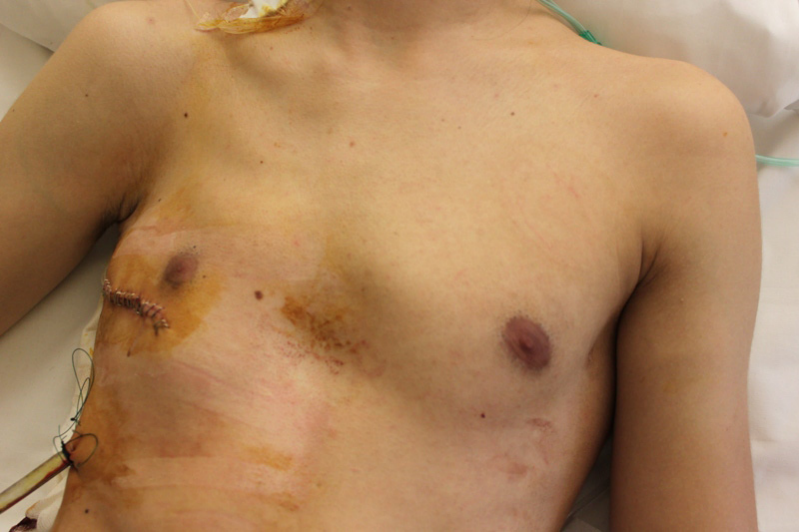
**Photograph of the affected hemithorax of one of the patients after the operation**.

Two young (aged 19 and 21 years old) male patients with Poland's syndrome were admitted (with an interval of 6 months from each other) to our department after their second episode of pneumothorax for which they had to be treated with the placement of closed chest tube thoracostomy. Thorax CT imaging revealed the presence of 5-6 bullae to the ipsilateral (right) with the syndrome lung (Figures 1 and 2). Both patients were submitted to surgery (bullectomy, lung apicectomy, partial parietal pleurectomy, and chemical pleurodesis with hyperosmotic 35% Dextrose solution) with total lung expansion and excellent results both on discharge and on the 3-month follow-up control. The patients' informed consent was obtained prior to their inclusion in this report.

## Conclusions-Discussion

Although the complex genetic and embryological mechanisms behind the genesis of developmental deformities are in most cases unknown, the knowledge of such clinical syndromes can always be useful for practicing physicians. In the current study we are reporting the cases of two young male patients with Poland's syndrome who were admitted with recurrent pneumothorax due to the presence of lung bullae affecting the ipsilateral to the syndrome side. Although more cases of the kind are needed to confirm lung bullous disease as a part or a variation of Poland's syndrome, one could say that, based on the current report and two similar published in the past, comprising 3 patients in total [5,6], augmented clinical suspicion for the occurrence of pneumothorax by patients with the syndrome and their attending physicians is justified. Whether this clinical suspicion also justifies the routine performance of thorax CT to all patients with Poland's syndrome is yet to be determined, based on each doctor's sense and also on future cases of the kind that will definitely correlate the syndrome with the presence of lung bullous disease.

## Consent

'Written informed consent was obtained from the patients for publication of this case report and accompanying images. A copy of the written consent is available for review by the Editor-in-Chief of this journal.'

## Competing interests

The authors declare that they have no competing interests.

## Authors' contributions

GT was a member of the surgical team and key author; DK coauthored the paper;

AT was a member of the surgical team and performed literature research; FT performed as an ophthalmologist ocular control in both cases and performed the related literature research; MD performed literature research and controlled linguistically the paper; AH was a member of the surgical team and controlled the paper; KK was the anesthesiologist in one of the cases and controlled the paper; KS was the anesthesiologist in on of the cases and controlled the paper; SL performed linguistic control and literature research; NT is the Head of the Department and made the final control to the paper. 'All authors read and approved the final manuscript'.
